# Application of ultrasound in avoiding radial nerve injury during elbow arthroscopy: a retrospective follow-up study

**DOI:** 10.1186/s12891-022-06109-8

**Published:** 2022-12-24

**Authors:** Xingtao Ge, Xinghua Ge, Chen Wang, Qinghua Liu, Bin Wang, Longgang Chen, Kai Cheng, Ming Qin

**Affiliations:** 1 Department of Orthopedics, Rizhao People’s Hospital, 276800 Rizhao, Shandong P.R. China; 2 Department of Neurosurgery, Rizhao People’s Hospital, 276800 Rizhao, Shandong P.R. China; 3 Department of Ultrasonography, Rizhao People’s Hospital, 276800 Rizhao, Shandong P.R. China

**Keywords:** Ultrasound, Elbow arthroscopy, Clinical outcomes, Radial nerve injury

## Abstract

**Background:**

A safe and effective technique for anterolateral portal placement in elbow arthroscopy is significant. We compared the outcomes of patients who underwent elbow arthroscopy using different ultrasound-assisted techniques.

**Methods:**

From May 2016 to June 2021 a retrospective analysis on all patients who underwent elbow arthroscopy in our department was performed. Patients were separated into three groups: non-ultrasound; preoperative ultrasound; and intraoperative ultrasound. The minimum follow-up period was 1 year. Nerve injuries, visual analog scale (VAS), Mayo elbow-performance score (MEPS), Disabilities of the Arm, Shoulder, and Hand Questionnaire (DASH), and range of motion (ROM) of the elbow were evaluated for comparison among the three groups pre- and post-operatively.

**Results:**

All 55 patients completed a 1-year follow-up: non-ultrasound (*n* = 20); preoperative ultrasound (*n* = 17); and intraoperative ultrasound (*n* = 18). There were 3 cases (15.0%) of transient radial nerve palsy in the non-ultrasound group. No nerve complications occurred in preoperative ultrasound and intraoperative ultrasound groups. The probability of postoperative radial nerve injury in the three groups was statistically different (*P* < 0.05). There was no significant difference in the VAS score, MEPS, DASH score, and ROM among the three groups at the follow-up evaluation (*P* > 0.05).

**Conclusion:**

Performing anterolateral portal placement during elbow arthroscopy with ultrasound-assisted techniques successfully avoided radial nerve injury.

## Background

Elbow diseases are prevalent in the population, and elbow arthroscopy is commonly performed in elbow surgery. [1, 2]. The development of technology and arthroscopic instruments has made elbow arthroscopy safe and effective for multiple elbow pathologies [3–5]. Due to the complex anatomic structure of the elbow joint, neurologic complications, including transient and permanent nerve injuries, remain a challenge for surgeons who perform elbow arthroscopy [6–8]. Direct trauma, especially portal placement during the manipulation procedure, is one of the common causes of nerve injuries during elbow arthroscopy [9, 10]. Previous studies have shown that the prevalence of neurologic injury in elbow arthroscopy ranges from 1.7 to 10.4%, with radial nerve injuries as high as 22% [11]. Establishing a safe anterolateral approach is crucial to avoiding radial nerve injuries [12–14]. Powell et al. [15] reported that map planning surgical access would benefit from preoperative sonography of the ulnar nerve. In addition, Ohuchi et al. [16] concluded that ultrasound-assisted posteromedial portal placement of the elbow joint avoids ulnar nerve injuries; however, reports on the use of assistive techniques to establish an anterolateral approach are limited.

Therefore, this study was conducted to compare the outcomes of patients undergoing elbow arthroscopy with different ultrasound-assisted techniques to aid clinicians in using safer and more effective technology for anterolateral portal placement to prevent radial nerve injuries.

## Methods

From May 2016 to June 2021, a total of 113 patients who underwent elbow arthroscopy were reviewed retrospectively, 55 patients of whom were enrolled. The inclusion criteria were as follows: Proliferation of osteophyte and synovium in elbow; limited flexion or extension of elbow joint; failed conservative treatment; accepting elbow arthroscopic debridement; and required anterolateral portal placement during the elbow arthroscopy procedure. The exclusion criteria were as follows: fracture history and previous elbow surgeries; elbow dislocation and ligamentous injuries; and neurologic injuries preoperatively. Initially, we completed the elbow surgery without ultrasound guidance. With improvements in the ultrasound equipment at our hospital, we gradually adopted preoperative ultrasound to identify the anterolateral portal. Then, we could determine the anterolateral portal under real-time ultrasound assistance with permission from the operating room staff. All surgical procedures were performed by a senior surgeon with 17 years of experience in joint surgery. Patients were divided into non-ultrasound, preoperative ultrasound, and intraoperative ultrasound groups based on different ultrasound techniques. Basic patient demographic information was collected, including age, gender, body mass index (BMI), surgical side, diabetes, and smoking history. All patients who met the criteria with anterolateral portal placement during surgery were documented, with particular attention to those patients who had postoperative radial nerve complications. This study was approved by the hospital ethics committee. Informed consent was obtained from all patients involved in the study.

### Surgical technique

All patients were placed in the supine position when undergoing elbow arthroscopy under general anesthesia. The shoulder was placed in 90° abduction and the elbow was placed in 90° flexion. After aseptic preparation and tourniquet application, the direct lateral portal (soft spot) placement of the elbow was first established during the operation, then the anterolateral portal placement of the elbow was established. Patients in the non-ultrasound group did not undergo ultrasound testing, thus the anterolateral portal placement in the elbow was established by palpation. The surgeon made skin incisions for the anterolateral portals before the insertion of any surgical instruments into the elbow compartment, and the anterolateral portal was identified by palpation during rotation of the forearm. In the preoperative ultrasound group, the surgeon identified the most suitable anterolateral portal according to the preoperative radial nerve map. The surgeon determined the anterolateral portal under real-time ultrasound assistance in the intraoperative ultrasound group. After establishment of the anterolateral portal, the surgeon performed all necessary procedures in the joint space. If requested by the surgeon, other portal placements were performed and the operative procedures were completed. Final debridement was done through all visible areas of the elbow joint, and the portals were thoroughly irrigated and closed with sutures. Compressive sterile dressings with bandages were applied to the elbow.

### Ultrasound technique

Ultrasound testing was performed by a senior radiologist with 13 years of experience. The radial nerve was visualized with a linear transducer. Patients accepted ultrasound testing in the pre- and intra-operative ultrasound groups.

**Fig. 1:**
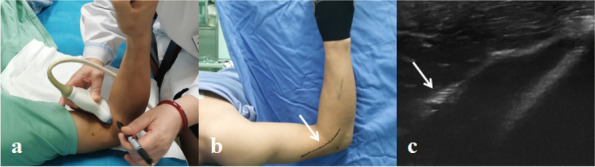
A 33-year-old male in the preoperative ultrasound group. **a** prior subcutaneous radial nerve demonstrating the transducer position and marking (dots) of the radial nerve during mapping; (**b**) the path of the radial nerve (white arrow) was drawn by a solid line; (**c**) the ultrasound images of the radial nerve (white arrow)

Patients in the preoperative ultrasound group underwent ultrasound assessment preoperatively to map the path of the radial nerve. Ultrasound testing was performed with the shoulder in 90° abduction and the elbow in 90° flexion to simulate the position during elbow arthroscopy. Thus, the radial nerve was also imaged and mapped in the supine position. Superficial structures were visualized under a 15-MHz frequency of standard ultrasound settings, and the nerve was imaged as a linear hypoechoic structure with fascicular architecture. The dots on the skin were marked at 1–2 cm intervals with a marking pen to trace the radial nerve. Then, a continuous line was drawn to complete the nerve map. The path of the radial nerve was from the distal humerus to the mid-forearm and the surgeon established anterolateral portal placement at the time of surgery as a reference, as shown in Fig. 1.

**Fig. 2:**
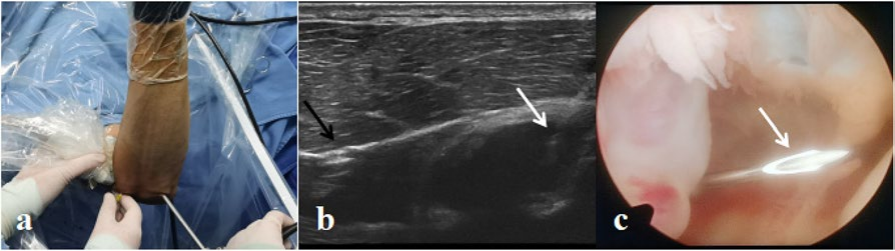
A 41-year-old male in the intraoperative ultrasound group. **a** Top view image of the right elbow joint with the patient in the supine position. A linear probe is held to view the radial nerve. A pilot needle is slowly inserted inside the anterolateral elbow joint. **b** The real-time ultrasound image of the radial nerve (black arrow) and pilot needle (white arrow). The needle is inserted into the anterolateral elbow joint. **c** Corresponding arthroscopic image obtained from the direct lateral viewing portal, showing insertion of the pilot needle (white arrow) into the anterolateral elbow joint

The ultrasound-assisted anterolateral portal was established intraoperatively for patients in the intraoperative group. Short- and long-axis images of the radial nerve were visualized with real-time ultrasound. Based on the image, a needle was inserted into the anterolateral elbow joint using a perpendicular approach. A skin incision was made close to the needle and deepened to the capsule. The anterolateral portal was completely built with insertion of the arthroscope, as shown in Fig. 2.

### Clinical assessment

We called patients back to evaluate the postoperative symptoms of a nerve injury every month, and patients could contact us at any time. The patients were asked about any disturbance of sensation or movement in the distribution of the nerves. Radial nerve injuries were of particular concern; finger, thumb, and wrist extension were tested in addition to sensation involving the dorsal aspect of the forearm. When patients presented with symptoms of a nerve injury, we used electromyography (EMG) to make a definite diagnosis. The radial nerve injury was identified as temporary nerve palsy if symptoms of the nerve injury resolved during the 12-month follow-up. The radial nerve injury was considered permanent if any optimal sensibility or function did not recover completely at a minimum follow-up duration of 12 months [17].

A visual analog scale (VAS) was used to determine pre- and post-operative pain. To evaluate clinical outcomes of elbows before and after surgery, two scales were used: the Mayo elbow-performance score (MEPS), consisting of pain, exercising, stability, and self-care ability; and the Disabilities of the Arm, Shoulder, and Hand Questionnaire (DASH), consisting of activity and symptoms. The questionnaires were performed before and after surgery at 6 and 12 months [18, 19]. Active range of motion (ROM), including extension-flexion and pronation-supination in the neutral position was measured with a goniometer [20]. The ROM values were also recorded before and after surgery at 6 and 12 months.

### Statistical analysis

All data were analyzed using SPSS 26.0 statistical software. Data measurements are expressed as the mean ± standard deviation (SD). An independent sample t-test was adopted to compare quantitative data between groups. Comparisons of clinical scores were performed using Analysis of Variance (ANOVA) at different time points. The difference between pre- and post-operative scores of each group was detected using a paired t-test. The chi-square test was used for categorical variables. A *P* < 0.05 was considered statistically significant.

## Results

Fifty-five patients met the inclusion criteria and completed the 12-month follow-up evaluation. There were 20 patients 53.7 ± 4.3 years of age in the non-ultrasound group. There were 17 patients 54.0 ± 6.2 years of age in the preoperative ultrasound group. There were 18 patients 52.9 ± 4.5 years of age in the intraoperative ultrasound group. There were no significant differences in demographic characteristics, such as age, gender, BMI, operative side, diabetes, and smoking among the three groups (Table 1).

**Table 1:** Demographic data

	Non-ultrasound	Preoperative ultrasound	Intraoperative ultrasound	*P*
Male/female	12/8	8/9	10/8	0.729
Age (year)	53.7 ± 4.3	54.0 ± 6.2	52.9 ± 4.5	0.815
BMI (kg/m^2^)	22.3 ± 2.7	23.4 ± 2.6	22.1 ± 2.9	0.361
Side of involvement (left/right)	9/11	7/10	8/10	0.853
Diabetes	2	3	2	0.770
Smoking history	2	3	1	0.513

Nerve palsies in the distribution region of the radial nerve occurred postoperatively. In the non-ultrasound group, there were 3 cases (15.0%) of transient radial nerve palsies (Table 2). No nerve complications occurred in the preoperative ultrasound group and intraoperative ultrasound group. The probability of postoperative radial nerve injury in the three groups was statistically different (*P* < 0.05).

**Table 2:** EMG results of radial nerve injuries

Radial nerve injuries	Side	Latency	Amplitude	Motor conduction velocity	Sensory conduction velocity
Incomplete injury	Left	Normal	Decrease	Normal	Normal
Incomplete injury	Right	Normal	Normal	Normal	Slowness
Incomplete injury	Right	Normal	Decrease	Slowness	Normal

**Fig. 3:**
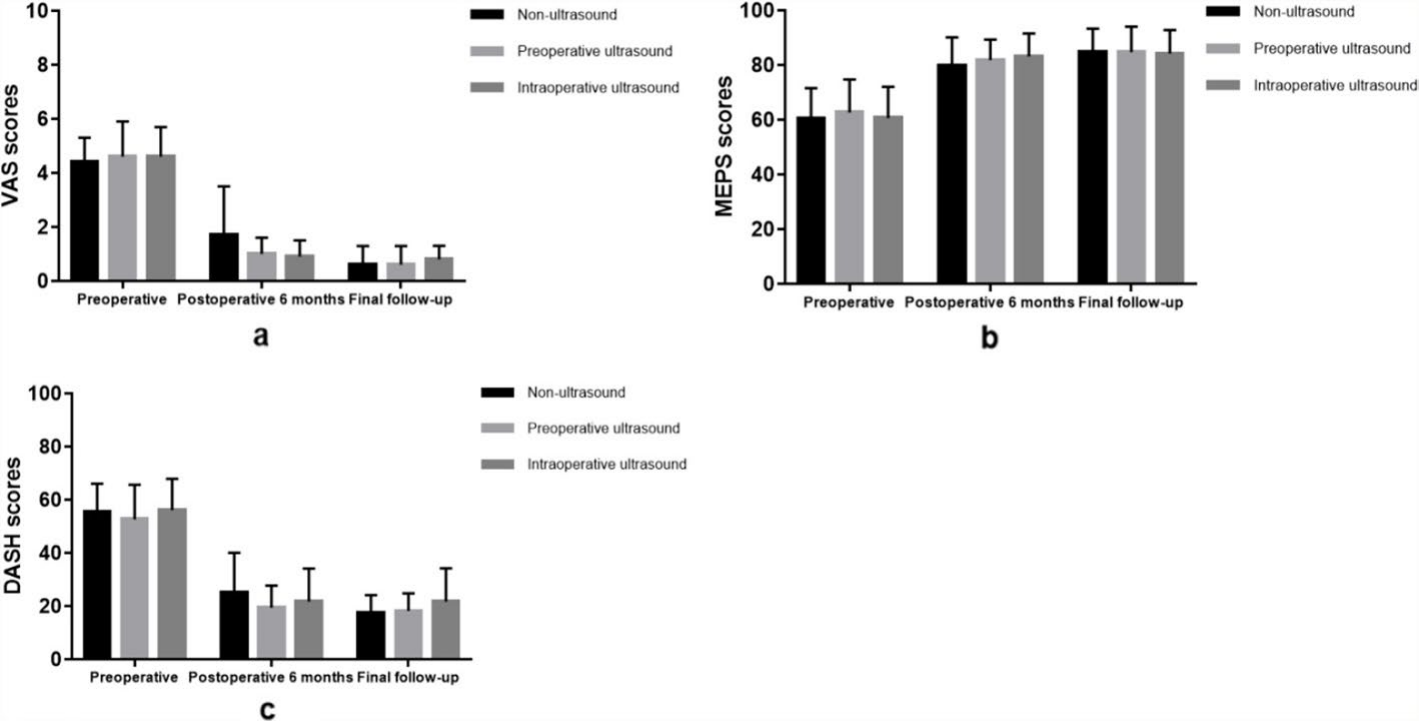
VAS (**a**), MEPS (**b**) and DASH (**c**) scores before and after the operation in the 3 groups

There was no significant difference in the VAS score (*P* = 0.871), MEPS score (*P* = 0.819) and DASH score (*P* = 0.675) among the three groups before the operation. Compared with the preoperative clinical scores, the VAS score, MEPS score and DASH score in the three groups were significantly improved after the operation (*P* < 0.05). The VAS score (*P* = 0.359), MEPS score (*P* = 0.662) and DASH score (*P* = 0.510) did not differ among the 3 groups at 6 months after the operation. There was no significant difference in VAS score (*P* = 0.178), MEPS score (*P* = 0.946), and DASH score (*P* = 0.821) among three groups at the final follow-up evaluation (Fig. 3).

**Fig. 4:**
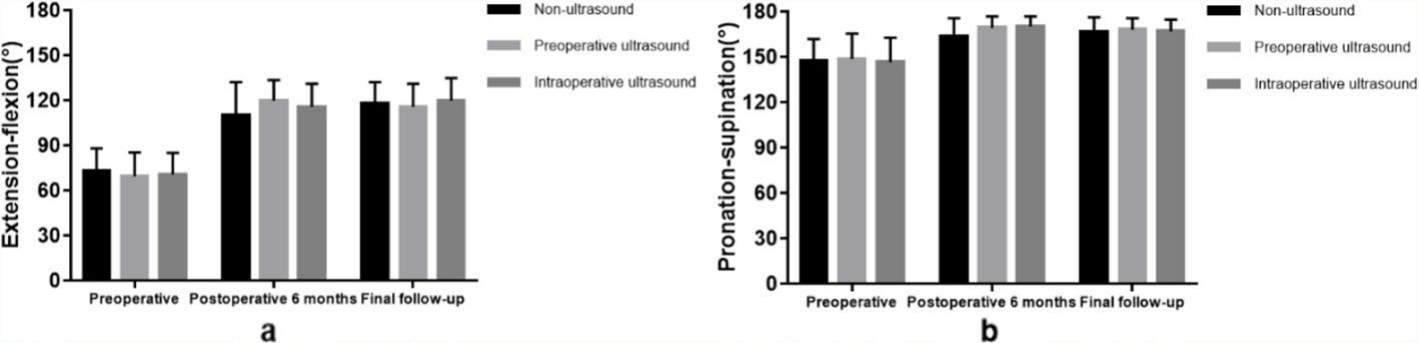
Extension-flexion (**a**) and Pronation-supination (**b**) values before and after the operation in the 3 groups

There was no significant difference in the extension-flexion (*P* = 0.779) and pronation-supination (*P* = 0.844) among the three groups before the operation. Compared with the preoperative clinical scores, the extension-flexion and pronation-supination in the three groups were significantly improved after the operation (*P* < 0.05). The extension-flexion (*P* = 0.421) and pronation-supination (*P* = 0.100) did not differ among the 3 groups at 6 months after the operation. There was no significant difference in the extension-flexion (*P* = 0.828) and pronation-supination (*P* = 0.951) among three groups at the final follow-up evaluation (Fig. 4).

## Discussion

In this study we compared the clinical outcomes of patients in three different groups. Based on the results, there were 3 cases of transient radial nerve palsies patients in the non- ultrasound group. But No nerve complications occurred in patients who accepted ultrasound guidance before or during the operation at the follow-up evaluation. In addition, the function scores including VAS, MEPS, and DASH scores, and ROM of the elbow had similar outcomes among the non-, pre- and intra-operative ultrasound groups at 6 and 12 months postoperatively.

The use of elbow arthroscopy for related elbow joint procedures has continued to increase over time, necessitating a greater assessment of the associated complications after elbow arthroscopy [21–23]. Jinnah et al. [11] reported that 222 nerve injuries occurred in 372 respondents; ulnar, radial, and posterior interosseous nerve injuries occurred in 38%, 22%, and 19% of the patients, respectively. These findings indicate that major nerve injuries after elbow arthroscopy are not rare occurrences. To the observations of general elbow arthroscopy, we also focused on the outcomes of patients accepting different forms of ultrasound guidance. Because of the high probability of radial nerve injuries, we mainly observed radial injuries after elbow arthroscopy. The nerve injuries in the non-ultrasound was the radial nerve in 15% of the patients. The probability of nerve injuries in our study was lower and the main injuries involved the radial nerve.

Previous studies [6, 7, 24] have demonstrated that the radial nerve is prone to injury during the anterolateral approach in elbow arthroscopy. The radial nerve is directly anterior to the radial head, and the anterolateral portal is located anterior to the articulation of the humeroradial joint [25]. Thaveepunsan et al. [26] reported a needle-and-knife technique for anterolateral portal placement during elbow arthroscopy. The anterolateral portal placement was also the focus in our study, and we suggested a safe and effective technique with ultrasound during surgery. We could directly see the radial nerve under the ultrasound image, and there were no complications using this technique. Using an ultrasound-assisted technique provides precise location of the nerve in the case of nerve variation.

Powell et al. [15] reported that preoperative mapping facilitates planning of surgical access. They preformed preoperative sonographic ulnar nerve mapping following various elbow operations. It was concluded that sonographic mapping of the ulnar nerve mitigates the potential inaccuracy of nerve palpation in a complicated postoperative elbow joint, and this technique may reduce the risk of ulnar nerve injury. Preoperative mapping under ultrasound technique was our preference, but we focused on the radial nerve. We drew the path of the radial nerve from the distal humerus to the mid-forearm as a reference during surgery. There was no patient with radial nerve palsy in the preoperative ultrasound group. Our study was a further investigation for the application of preoperative mapping under ultrasound, and we concluded that preoperative radial nerve mapping under ultrasound is safe and effective.

Ohuchi et al. [16] presented a technique applicable to portal placement of the elbow using an ultrasound-assisted method. However, they did not perform this technique in a large number of cases. Although we used the ultrasound-assisted technique during elbow arthroscopy, we focused on the anterolateral portal placement of the elbow joint. Portal placement with an ultrasound-assisted technique in elbow arthroscopy is a safe surgical method. Although the application of ultrasound in elbow arthroscopy may add surgical time and preoperative preparation, this method is a good way to avoid nerve injuries.

Hackl et al. [17] pointed out that the radial nerve shifts due to flexion and joint insufflation. The distance of the median and radial nerves to osseous landmarks doubles from extension to 90°flexion and triples after joint insufflation. In our study, preoperative markers of the radial nerve could shift according to Hackl et al. But in the results, there was no nerve injury in preoperative ultrasound group. We thought that although the radial nerve shifts due to flexion and joint insufflation, drawing path of the radial nerve preoperatively could also provide a valuable reference.

The VAS score has been used to evaluate pain, and elbow function can be assessed by MEPS and DASH scores, subjectively and objectively [27–29]. The ROM of the elbow in the neutral position reveals significant values to evaluate elbow function. Previous studies have demonstrated that arthroscopic elbow surgery leads to improved ROM and health-related quality of life in elbow disorders [30–32]. In our study, there were no significant differences in VAS and functional scores among the three groups at the 6 and 12-month follow-up evaluation.

The surgery was performed without ultrasound guidance at initial. then we gradually performed elbow arthroscopy with ultrasound assistance. All surgical procedures were performed by a senior surgeon with 17 years of experience in joint surgery. The surgeon with extensive knowledge of elbow anatomy might reduce the influence of the surgeon’s experience on the operation complication.

Although evaluation of patient outcomes in our study was more comprehensive, this study had several limitations. Because of the strict inclusion criteria, the study population was small. In addition, this was a retrospective study with all the associated biases, and future prospective randomized controlled studies with long-term follow-up are warranted to confirm our findings.

## Conclusion

Performing anterolateral portal placement with an ultrasound-assisted techniques is a safe and efficient method to avoid radial nerve injuries in elbow arthroscopy; however, the present study showed good clinical outcomes after elbow arthroscopy at the final follow-up evaluation

## Data Availability

All data generated or analyzed during this study are included in this article.

## Abbreviations

VAS: Visual analog scale
MEPS: Mayo elbow-performance score
DASH: Disabilities of the Arm, Shoulder, and Hand Questionnaire
ROM: Range of motion
BMI: Body mass index
EMG: electromyography
SD: standard deviation
ANOVA: Analysis of Variance
